# Relationship between Energy Expenditure Related Factors and Oxidative Stress in Follicular Fluid

**Published:** 2014-07-08

**Authors:** Ashraf Kazemi, Fatemeh Ramezanzadeh, Mohammad Hosein Nasr Esfahani, Ali Akbar Saboor-Yaraghi, Saharnaz Nejat Nejat, Abbas Rahimi-Foroshani

**Affiliations:** 1 Nursing and Midwifery Care Research Center, Faculty of Nursing and Midwifery, Isfahan University of Medical Sciences, Isfahan, Iran; 2 School of Nursing and Midwifery, Tehran University of Medical Sciences, Tehran, Iran; 3 Vali-e-Asr Reproductive Health Research Center, Imam Khomeini Hospital Complex, Tehran University of Medical Sciences, Tehran, Iran; 4 Animal Core Facility at Reproductive Biomedicine Research Center, Royan Institute for Biotechnology, ACECR, Tehran, Iran; 5 Department of Nutrition and Biochemistry, School of Public Health, Tehran University of Medical Sciences, Tehran, Iran; 6 Department of Epidemiology and Biostatistics, Knowledge Utilization Research Center, School of Public Health, Tehran University of Medical Sciences, Tehran, Iran

**Keywords:** Energy Expenditure, Calorie Intake, Physical Activity, Oxidative Stress, Follicular Fluid

## Abstract

**Background:**

This study evaluated the impact of body mass index (BMI), total calorie intake and physical activity (PA) as energy expenditure related factors on oxidative stress (OS) in follicular fluid (FF).

**Materials and Methods:**

This prospective study conducted on 219 infertile women. We evaluated patients’ BMI, total calorie intake and PA in their assisted reproduction treatment cycles. Malondialdehyde (MDA) and total antioxidant capacity (TAC) in pooled FF at oocyte retrieval were additionally assessed.

**Results:**

There was no relation between OS biomarkers to total calorie intake and PA. The TAC levels in FF adjusted for age, duration of infertility, etiology of infertility, number of used gonadotrophin and PA showed a positive relation to BMI (p=0.001). The number of used gonadotrophin and PA had a negative relation to duration of infertility (p=0.03) and anovulation disorder as an etiology of infertility. The MDA level in FF had a positive association with anovulation disorder as the etiology of infertility (p=0.02). MDA in FF was unaffected by BMI.

**Conclusion:**

Increasing age, BMI and PA do not affect OS in FF. In women with longtime infertility and those with anovulation disorder as an etiology of infertility, decreased potent antioxidant defense in the follicular microenvironment may contribute to ovarian function. Therefore antioxidant supplements may be beneficial for these groups of women.

## Introduction

Follicular fluid (FF) represents a very important microenvironment. It is a metabolically active system that plays a critical role in constituting a complex, regulated ovarian microenvironment. This environment, in addition to granulosa cells, cytokines and macrophages all produce reactive oxygen species (1). Reactive oxygen species target the macromolecules in cells such as lipids, proteins and nucleic acids, causing peroxidative damage. Under normal conditions, scavenging molecules known as antioxidants prevent overproduction of these toxic products. When the balance between free radicals and antioxidants is tipped towards an overabundance of free radicals, oxidative stress (OS) occurs (2) which is the cause of molecular damage to vital structures and functions (3). Any disturbances in these organelles can lead to profound problems such as ATP generation, which is essential for cellular function (4). The impact of OS in FF on the female reproductive system has been evaluated in different studies.

Several studies have provided evidence that OS in antral follicle and in media culture cause deleterious effects in human oocytes and embryos *in vitro* (5-11). It is of interest to find factors related to life style that decreases OS in the oocyte environment. However, to our knowledge, there is no study which has evaluated the impact of life style on OS in human FF.

In the literature there are numerous studies that have considered life style to be a cause of OS. The prominent risk factors for increased lipid peroxidation are smoking (12), obesity (13-15) and sedentary life style (16) which can deplete a potent antioxidant defense. Although the expression profiles of the transcripts of the antioxidant enzymes in human oviducts and oocytes have also been shown (17), some non-enzymatic antioxidants in the FF originate from external sources. Decreasing antioxidants in plasma may be reflected in the follicular microenvironment and it prepares this environment for the peroxidative process. In addition, assisted reproduction techniques provide an *in vitro* model to study the factors that affect OS in the oocyte environment. Therefore, the present study seeks to determine the impact of a number of factors related to life style including calorie intake, physical activity (PA) and body mass index (BMI), on OS in FF.

## Materials and Methods

In this prospective observational study enrolled women who underwent assisted reproduction treatment from July 2010 to April 2011 at Isfahan Fertility and Infertility Center. This study was approved by the Institutional Review Board and Ethics Committee of Tehran University of Medical Sciences.

Inclusion criteria were women 18-40 years of age who used their own oocytes (autologous) for fertility treatments. Male factors according to World Health Organization criteria (18), considerable change in dietary regimen over the previous three months and during assisted reproduction cycle, in addition to the presence of systemic diseases such as hepatic diseases, endocrine disorders and metabolic disorders, were considered as exclusion criteria. Informed consent was obtained from all subjects.

On day three of a spontaneous menstrual cycle, we calculated BMI by dividing weight in kilograms by height in meters squared.

A validated semi-quantitative food frequency questionnaire (19) was used to measure the calorie intake over the previous three months. The questionnaires were completed by participants. Nutritionist software version IV (Nutritionist IV, Version 3.5.2) was used to calculate the daily energy intake.

PA was measured using the original International Physical Activity Questionnaire. This questionnaire assessed energy expenditure in total, by moderate and vigorous intensity (20) as well as MET values and formula for computation of MET-minutes (21). Age and etiology of infertility were considered as influential factors.

The long protocol that involved a gonadotrophin-releasing hormone (GnRH) agonist and human menopausal gonadotrophin (hMG) administration was consistent. Follicular maturation was monitored by ultrasound examination. At oocyte retrieval, fluid from an average of one to 5 follicles that were 16-mm diameter or larger was pooled. Samples that appeared blood stained or without oocytes were discarded. The samples were immediately centrifuged at 300 rpm for 17 minutes, after which 2 ml of the supernatant was stored in microtubes at -70˚C for a maximum of 2 weeks until analyses for malondialdehyde (MDA) and total antioxidant capacity (TAC).

FF lipid peroxidation and antioxidant defense activity were measured as the levels of MDA and TAC. Follicular MDA was determined by the 2-thiobarbituric acid reactive substances (TBARS) method (22). The results were expressed as micromoles MDA/liter of FF (μmol/l). TAC was measured in the FF using an enhanced chemiluminescence assay described previously (1). The results were expressed as molar Trolox equivalents.

Statistical analysis was conducted using SPSS version 13.0 (SPSS, Chicago, IL, USA). The data were analyzed using multivariable liner regression analysis (adjusted for age, etiology of infertility, duration of infertility and number of used gonadotrophin and passive smoking). All variables were entered in the model and unstandardized regression coefficients (B) with 95% confidence interval (95% CI) were reported. The etiology of infertility and passive smoking were used as qualitative with a dummy code. Also, ANOVA was used for comparing age, BMI, PA, etiology of infertility, duration of infertility and number of used gonadotrophin between women whom FF biomarkers were assessed and those with FF were not available. P-values of <0.05 were considered significant.

## Results

Of the 240 eligible women (18-40 years of age) who participated, 4 individuals withdrew from the assisted reproduction program prior to study completion. FF biomarkers for 17 participants were not assessed because their follicles did not have oocytes or the FF sample volumes were insufficient. The age, BMI, PA, etiology of infertility, duration of infertility and number of used gonadotrophin did not differ between women whose FF biomarkers were assessed and those whose markers were unavailable (data not shown).

The baseline data and characteristics of 219 patients with the mean age of 31.54 years are presented in table 1.

**Table 1:** Characteristics of subjects


Variables	Mean (SD) or %

**Age (years)**	31.54 (6.20)
**Duration of infertility**	7.42 (5.14)
**No. of used gonadotrophin**	40.54 (17.06)
**Etiology of infertility (%)**	
** PCOS **	29.7%
** Endometriosis**	18.2%
** Anovulation**	15.3%
** Others**	36.8%
**Total calorie intake (kcal)**	2167.8 (717.2)
**PA (METs-minutes-day)**	31.82 (5.51)
**BMI (kg/m^2^)**	26.6 (4.3)
**Active smoker (%)**	0.00%
**Passive smoker (%)**	30.10%
**MDA (µmol/lit)**	0.98 (0.28)
**TAC (molar Trolox equivalents)**	1987.73 (354.08)


SD; Standard deviation, PA; Physical activity, BMI;Body mass index, PCOS; Polycystic ovarian syndrome, MDA; Malondialdehyde and TAC; Total antioxidant capacity

**Table 2:** Relation between energy expenditure related factors and oxidative stress (OS) in follicular fluid (FF)


Model A: Outcome MDA R^2^=0.050							
		Unstandardized coefficient			Standardizedcoefficient	Sig	95% Confidence interval (95% CI)			
		B	SE	Beta		Lower	Upper

**Confounders**	**Age**	-0.01	0.004	0.06	NS	-0.01	0.02
	**Duration of infertility**	0.01	0.01	0.01	NS	-0.18	0.16
	**Number of used gonadotrophin**	0.01	0.01	0.09	NS	-0.01	0.01
	**Passive smokers**	0.01	0.03	0.05	NS	-0.02	0.04
	**Etiology of infertility**
	**PCOS**	-0.11	0.1	0.08	NS	-0.23	0.09
	**Endometriosis**	-0.04	0.05	0.07	NS	-0.17	0.09
	**Anovulation**	0.13	0.05	0.17	0.02	0.02	0.25
	**Other**	-0.14	0.04	-0.09	0.02	-0.25	-0.03
**Independence**	**PA**	0.01	0.01	0.02	NS	-0.18	0.16
	**BMI**	-0.06	0.01	0.03	NS	-0.01	0.02
	**Total calorie intake**	-0.36	0.12	0.08	NS	-1.49	0.78
**Model B: Outcome TAC R^2^=0.075**							
		**Unstandardized coefficient**		**Standardized coefficient**	**Sig**	**95% Confidence interval (95% CI)**	
		**B**	**SE of B**	**Beta**		**Lower**	**Upper**
**Confounders**	**Age**	-3.78	4.66	-0.05	NS	-13.05	5.48
	**Duration of infertility**	-11.3	5.14	-0.18	0.02	-21.38	-0.12
	**Number of used gonadotrophin**	-1.78	1.55	-0.07	NS	-4.77	1.04
	**Passive smokers**	-2.8	54.28	0.01	NS	-3.2	1.96
	**Etiology of infertility**						
	**PCOS**	10.4	62.12	-0.01	NS	-123.86	146.79
	**Endometriosis**	24.3	84.464	-0.06	NS	-118.89	163.62
	**Anovulation**	58.1	73.41	0.06	NS	-89.91	206.07
	**Other**	-53.7	73.14	-0.07	NS	-201.75	94.38
**Independence**	**PA**	1.31	0.02	0.04	NS	-21.46	21.08
	**BMI**	17.3	6.06	0.24	0.002	6. 52	30.07
	**Total calorie intake**	-0.02	0.03	-0.04	NS	-0.08	0.05


MDA; Malondialdehyde, TAC; Total antioxidant capacity, PCOS; Polycystic ovaries syndrome, PA; Physical activity, BMI; Body mass index, Sig; Significance, NS; Non-significant and SE; Standard error.

According to regression analysis when adjusted for age, etiology of infertility, duration of infertility and the number of used gonadotropin, there was no relation between MDA levels in FF and BMI (B=-0.06, CI=-0.01- 0.02), PA (B=0.01, CI=-0.18- 0.16) and total calorie intake (B=-0.36, CI=-1.49- 0.78). There was a positive relation between TAC level and BMI (B=73.30, CI=14.15- 47.31, p=0.001), but there was no relation to PA (B=1.31, CI=-212.46- 215.08) and total calorie intake (B=-126.05, CI=-20.54- 272.64).

Of the influential factors, we observed a positive relation between anovulation disorder (B=0.13, CI=0.02- 0.25, p=0.02) and MDA levels in FF. Other infertility etiologies (tubal factor, hypothalamic amenorrhea and unexplained factor) had a negative association (B=-0.14, CI=-0.25- 0.03, p=0.02) to MDA levels in FF.

The mean MDA level (F=1.20, p=0.31) and the TAC levels (F=0.29, p=0.84) did not differ in the four groups based on etiology of infertility. The MDA levels (B=-0.01, CI=-0.01- 0.02) and the TAC levels (B=-3.78, CI=-13.05- 5.48) in FF were not related to age.

## Discussion

This study presents the first clinical evidence based on human assisted reproduction for a relation between life style factors and OS in the follicular microenvironment, considering PA and calorie intake on assisted reproduction parameters as factors of energy expenditure.

Several lines of evidence suggest the importance of age in a decrease in the efficiency of the follicular antioxidant defence system (23, 24). Previous studies have revealed an association between reproductive aging to an increase in free radicals (5) and a decrease in catalase in granulosa cells from preovulatory follicles (25, 26). But, in the current study, there was no relation between MDA and TAC levels in FF and age. Similar to our findings, Liu and Li (27) found no association between MDA level and age. Duration of infertility, in contrast to age, had a negative relation with potent antioxidant defense in FF. This finding has suggested that decreases in or depletion of antioxidants in the follicular environment may followed by reproduction failure. Clinically, antioxidant supplements may improve assisted reproduction outcome in women with longtime infertility.

Another finding of the present study revealed that independent to age, etiology of infertility, number of used gonadotrophin, PA and calorie intake, the TAC levels in FF was positively related to BMI. This was consistent with a previous finding. Previously it was explained that dead granulosa cells could theoretically contribute to the passive release of antioxidants into the FF and contribute to a negative outcome of *in vitro* fertilization therapy (28). The increasing number of apoptotic cells formed in fresh follicle harvests from obese women compared with normal-weight patients (29) might explain this finding.

The increased TAC levels in FF in women with higher BMI might refer to antioxidant effect of estradiol (30, 31) and to increased estradiol levels in FF in this group due to ovarian stimulation (32). An increased intake of foods with high TAC levels in women with higher BMI (33) might be another explanation for this finding. According to Ozkaya and Nazıroglu multivitamin and mineral supplementation in serum and FF of women undergoing IVF might strengthen the antioxidant defense system by decreasing OS (34).

Numerous studies have demonstrated that metabolic and hormonal changes, followed by obesity could induce systemic OS (13-15), deplete potent antioxidant defense in plasma and decrease transfer of micronutrients that have antioxidant effects. Therefore, an increase in food with antioxidant effects in women with higher BMI is not the main cause for increased TAC levels in FF in these women.

It was demonstrated that follicle-stimulating hormone stimulated from glutathione synthesis suppressed the production of reactive oxygen species and decreased the rate of apoptosis in cultured follicles (35). Although we observed no relation between the number of used gonadotrophins and OS markers the longtime increased level of endogenous gonadotrophin in overweight women might lead to increased production of antioxidants in the follicular environment.

In contrast to TAC, there was no relation between MDA level in FF and BMI. However, the increased TAC level in overweight women might protect the follicular environment from lipid peroxidation.

Another finding of the present study revealed that OS biomarkers were not related to total calorie intake and PA. Therefore the benefit effect of PA on reproduction (36, 37) could be due to other mechanisms.

The present study indicated that the MDA level in FF did not differ in women with different etiologies of infertility, but the MDA level in FF, independent of energy expenditure related factors, had a positive relation to anovulation.

This finding suggested that OS in follicular environment or OS-induced factors might contribute to anovulation and infertility. A negative relation between other etiologies with normal ovarian function (such as tubal factor and primary amenorrhea) and MDA level in FF might power this suggestion. Therefore, antioxidant supplements might benefit women with anovulation disorder.

We did not observe any relation between PCOS and endometriosis and OS in FF. Our observations were consistent with previous studies that reported OS in plasma and FF was not related to etiology of infertility (28, 38). According to Bausenwein, obesity elevated oxidized low-density lipoprotein levels in FF were associated with obesity itself, not with hormonal characteristic of PCOS (39).

In the present study women with systemic disorders such as hyperlipidemia and insulin resistance were excluded. The adverse effect of obesity on OS in FF might link with these disorders.

## Conclusion

This study demonstrated that age and BMI independent of PA, calorie intake and etiology of infertility did not have important roles in inducing OS in the follicular environment. The duration of infertility was shown to have a stronger positive relation with decreased potent antioxidant defense in FF. Among etiologies of infertility, we have shown that anovulation was related to potent antioxidant defense in the follicular environment. Antioxidant supplements may be helpful for this group.
